# Towards Machine Learning-Aided Lung Cancer Clinical Routines: Approaches and Open Challenges

**DOI:** 10.3390/jpm12030480

**Published:** 2022-03-16

**Authors:** Francisco Silva, Tania Pereira, Inês Neves, Joana Morgado, Cláudia Freitas, Mafalda Malafaia, Joana Sousa, João Fonseca, Eduardo Negrão, Beatriz Flor de Lima, Miguel Correia da Silva, António J. Madureira, Isabel Ramos, José Luis Costa, Venceslau Hespanhol, António Cunha, Hélder P. Oliveira

**Affiliations:** 1 INESC TEC—Institute for Systems and Computer Engineering, Technology and Science, 4200-465 Porto, Portugal; up201704832@edu.icbas.up.pt (I.N.); joana_morgado09@hotmail.com (J.M.); mafalda.m.oliveira@inesctec.pt (M.M.); joana.v.sousa@inesctec.pt (J.S.); joao.p.fonseca@inesctec.pt (J.F.); acunha@utad.pt (A.C.); helder.f.oliveira@inesctec.pt (H.P.O.); 2 FCUP—Faculty of Science, University of Porto, 4169-007 Porto, Portugal; 3 ICBAS—Abel Salazar Biomedical Sciences Institute, University of Porto, 4050-313 Porto, Portugal; 4 CHUSJ—Centro Hospitalar e Universitário de São João, 4200-319 Porto, Portugal; claudiaasfreitas@gmail.com (C.F.); eduardo.negrao@gmail.com (E.N.); beatrizflordelima@hotmail.com (B.F.d.L.); miguel.ncds@gmail.com (M.C.d.S.); antonio.madureira@chsj.min-saude.pt (A.J.M.); radiologia.hsj@gmail.com (I.R.); hespanholv@gmail.com (V.H.); 5 FMUP—Faculty of Medicine, University of Porto, 4200-319 Porto, Portugal; jcosta@ipatimup.pt; 6 FEUP—Faculty of Engineering, University of Porto, 4200-465 Porto, Portugal; 7 i3S—Instituto de Investigação e Inovação em Saúde, Universidade do Porto, 4200-135 Porto, Portugal; 8 IPATIMUP—Institute of Molecular Pathology and Immunology of the University of Porto, 4200-135 Porto, Portugal; 9 UTAD—University of Trás-os-Montes and Alto Douro, 5001-801 Vila Real, Portugal

**Keywords:** computer-aided decision, learning models, CT scan, lung cancer

## Abstract

Advancements in the development of computer-aided decision (CAD) systems for clinical routines provide unquestionable benefits in connecting human medical expertise with machine intelligence, to achieve better quality healthcare. Considering the large number of incidences and mortality numbers associated with lung cancer, there is a need for the most accurate clinical procedures; thus, the possibility of using artificial intelligence (AI) tools for decision support is becoming a closer reality. At any stage of the lung cancer clinical pathway, specific obstacles are identified and “motivate” the application of innovative AI solutions. This work provides a comprehensive review of the most recent research dedicated toward the development of CAD tools using computed tomography images for lung cancer-related tasks. We discuss the major challenges and provide critical perspectives on future directions. Although we focus on lung cancer in this review, we also provide a more clear definition of the path used to integrate AI in healthcare, emphasizing fundamental research points that are crucial for overcoming current barriers.

## 1. Introduction

Lung cancer is a disease that involves the accumulation of multiple genetic mutations and epigenetic changes, which results in an out-of-control cell proliferation that disrupts regular cells. Lung cancer is the leading cause of cancer-related fatalities worldwide, accounting for about 1.6 million deaths per year [1,2]; it is the second most common cancer diagnosis, comprising a total of 13% of new cancer cases each year [3]. Age is a risk factor for lung cancer [4], due to biologic factors, including DNA damage (over time) and telomere shortening. Smoking is the primary “agent” in the development of lung cancer, responsible for about 80% of lung cancer-related deaths [5]. Men and women who smoke are 23% and 13%, respectively, more likely to develop lung cancer compared to never-smokers [6]. The risk of being diagnosed with lung cancer, due to tobacco consumption, varies in ethnic groups, e.g., compared to white people, African Americans and native Hawaiian smokers are shown to be at a greater risk of developing lung cancer, with the highest incidences and death rates. Latino and Japanese American smokers are less likely to develop the disease and present the lowest cancer-specific mortality [1,6]. Accumulating evidence supports that genetic factors are also risk factors for lung cancer [7]. Recently, several novel lung cancer susceptibility genes, including those on chromosomes *6q23-25* and *13q31.3*, were identified by large-scale genome-wide association studies as being associated with lung cancer risk, particularly in never-smokers, who account for 25% of lung cancer patients worldwide [8,9]. Furthermore, an individual who has a positive family history of lung cancer has a 1.7-fold increased risk of developing lung cancer [9,10,11]. Lung cancer in never-smokers has been associated with genetic factors, as well as occupational exposures to lung carcinogens, exposure to ionizing radiation, and a poor diet [1,5,9,12].

**Figure 1:**
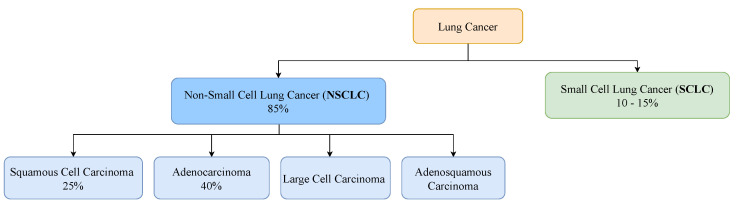
Prevalence of the major histological subtypes of lung cancer: non-small cell lung cancer and small cell lung cancer [13,14,15].

Lung cancer can be classified into two major histological subtypes: non-small cell lung cancer (NSCLC) and small cell lung cancer (SCLC). NSCLC accounts for about 85% of all lung cancer cases and presents a 25% chance of a 5-year-survival [13]. Adenocarcinoma and squamous cell carcinoma are its two major histologic types, accounting for about 40% and 25% of lung cancers, respectively [14]. SCLC is the lung cancer type that tends to spread the fastest, accounting for 10% to 15% of all lung cancers [15]. Individuals who have this type of lung cancer present a 7% chance of a 5-year-survival [13]. Figure 1 represents the distribution of the main histological subtypes.

Computed tomography (CT) is the most useful imaging modality used for lung cancer management, including diagnosis, staging, treatment planning, and treatment response evaluation [16,17,18,19]. CT is the recommended screening test for lung cancer; but confirmation of the malignancy and characterization of the nodule are traditionally conducted via a biopsy, which is an invasive and risky procedure for the patient that can lead to some clinical complications. Recently, non-invasive, fast, and easy-to-use techniques, such as computer-aided diagnosis (CAD) based on CT scans, have been developed for lung cancer characterization, to improve the accuracy of diagnosis, determine the most appropriate treatment for each subject and, consequently, decrease the mortality rate of patients battling lung cancer [20,21,22]. Since imaging is already regularly repeated during treatment, it has the potential to continuously supervise therapy and monitor the rise and growth of the disease or its response to therapy.

In this review, we present the main advances made in CADs dedicated to lung cancer diagnosis, characterization, and management; we also discuss the limitations of each part of the pipeline (e.g., screening and treatment assessments) and suggest directions for future developments. The primary focus of this work is on CAD systems that could be of help to clinicians in cancer management—from detection to treatment. We provide a deep summary of the state-of-the-art CADs. Due to the relevance of CT on lung cancer assessment, the CADs selected here are dedicated to this type of medical image data. We searched the following databases: Embase, PubMed, Cochrane Library, the Institute of Electrical and Electronics Engineers (IEEE), Scopus, Web of Science, conference proceedings, and the ACM Digital Library. For each section, additional keywords are used/detailed, as described during the review. This review begins with an overview of the clinical proceedings that comprise lung cancer clinical routines. Due to the relevance of the main biomarkers used to shape treatment plans, this work covers the biological pathways and elements that explain the importance of these biomarkers and the target therapies that have been (or are being) developed. A global overview of the clinical routines and biological elements involved in lung cancer genesis and development will help one understand the need (and impact) of CADs. This work reviews the CADs that have been (are being) developed to help clinicians in lung cancer management; it covers all stages of the clinical evaluations, and presents directions for the future, which are crucial for integration of CADs into clinical settings.

## 2. Clinical Pathway for Lung Cancer

The clinical pathway for lung cancer consists of the following main steps: screening, diagnosis, and treatment plan development [23]. The process of diagnosis begins with an initial evaluation, and it is followed by an analysis of tissues collected in the biopsy for the cancer confirmation, characterization, and staging. The treatment plan will consider the diagnosis and the patient’s functional status.

### 2.1. Screening

Screening involves testing an asymptomatic individual for a disease. Lung cancer typically does not cause signs or symptoms in its earliest stages; these only occur when the disease is advanced. Thus, screening exams are the most powerful tools for early detection. A total of 65% of patients are diagnosed when the disease has already reached the metastatic stage; these individuals have a 6% chance of 5-year-survival [24]. Only 17% of cases are diagnosed in a local state; in these cases, the 5-year survival rate increases to 59% [13]. The US national lung screening trial (NLST) and NELSON (two randomized controlled trials of low-dose CT (LDCT)-based lung cancer screenings in high-risk populations) showed evidence of a statistically significant mortality reduction in patients [19,25]. CT exams are recommended for adults aged 50 to 80 who have 20 packs-a-year smoking histories and who currently smoke or have quit within the past 15 years [26].

### 2.2. Diagnosis

#### 2.2.1. Initial Evaluation

The most common lung cancer symptoms are chronic cough, repeated respiratory infections, fatigue, hemoptysis, shortness of breath, hoarseness, and chest pain [27]. The initial evaluation should include a careful analysis of risk factors for lung cancer, prior history of cancer, evaluation of comorbidities, functional status, and overall health status [23]. All patients suspected of having lung cancer, who are undergoing initial evaluations, will require imaging studies. CT is the primary imaging exam performed to assess the existence of nodules and eventually lung cancer. The nodule size, nodule growth rate, and spiculation of the nodule anatomical margins are the main radiological predictors of malignancy risk [28]. The tissue biopsy will confirm the disease.

#### 2.2.2. Tissue Biopsy

Tissue biopsy is the current standard procedure for lung cancer classification, consisting of an assessment technique that includes several methods, such as fine needle aspiration, bronchoscopy, endobronchial ultrasound, mediastinoscopy, thoracentesis, thoracoscopy, and electromagnetic navigation. Bronchoscopy is the main diagnosis procedure, with flexible bronchoscopy being more useful for central lesions and navigational bronchoscopy displaying higher sensitivities for peripheral lesions [29,30]. Percutaneous approaches include transthoracic needle aspiration (TTNA) or needle/core biopsy (TTNB) of the primary tumor. The samples collected will be analysed in immunohistochemical stains and molecular tests in order to assess the mutation status of predominant oncogenes and identify the main biomarkers of the tumor, supporting precision medicine [31].

Despite a tissue biopsy being considered a relatively safe procedure, it is not free of complications since the invasive nature of a tissue biopsy limits its use, particularly in patients with inaccessible tumor sites or when repeated biopsies are needed [32]. Moreover, tissue biopsies have some limitations related to tumor heterogeneity, since a single biopsy may not represent the complexity of the entire tumor and its genetic alteration. Thus, there is an inability to carry out a complete therapeutic decision and prognosis, which are the main issue in clinical practices [29,30]. Therefore, although recommendations are clear about the need for a biopsy, its invasive nature limits repetition for treatment response evaluations [33].

#### 2.2.3. Liquid Biopsy

A liquid biopsy is a non-invasive, safe, and accessible technique that allows the detection of tumor cells or tumor-derived products in body fluids. Liquid biopsies consist of the analysis of circulating tumor cells and/or circulating tumor DNA (ctDNA) molecules employing simple tests on body fluid samples. A liquid biopsy may be viewed as a key strategy to improve lung cancer early diagnosis—either alone or as complementary data for imaging findings. Other clinical applications consist of patient stratification, therapeutic decisions, and disease monitoring [29,30]. Liquid biopsies are shown to be useful in the management of NSCLC in clinical practices. This approach overcomes both spatial and temporal tumor heterogeneity problems and enables repeatable evaluations of cancer patients while reducing the inherent risks and discomfort of tissue biopsies [34].

Several liquid biopsy-derived biomarkers have been identified, such as circulating tumor cells, circulating cell-free DNA, circulating micro-RNAs, tumor-derived exosomes, and tumor-educated platelets [29,30]. Evidence indicates that a liquid biopsy can be applied to dynamically evaluate resistance mutations during treatment with epidermal growth factor receptor (*EGFR*) and anaplastic lymphoma kinase (*ALK*) inhibitors [35]. However, there are limitations, i.e., related to low sensitivity for the detection in early stage tumors and, consequently, less utility in clinical practices [36,37]. For early stages of lung cancer, when the cancer biomarkers have very low values, the available biomarkers display significant proportions of false negatives or a need for a confirmatory tissue biopsy. Thus, the development of more sensitive and specific assays must occur in the following years to allow its standard use in clinical practice. There is also the possibility of combining different biomarkers and other diagnostic techniques, such as imaging techniques, although more robust studies are required to define the best combinations and to validate the clinical role of liquid biopsy in the screening or diagnosis of lung cancer [29,30].

#### 2.2.4. Staging

For confirmed malignant cases, it is important to determine the extension of cancer (cancer staging) and identify additional pathologies that can influence the treatment plan of the patient. The type of cancer is characterized in a triple form, using cytology and histology, immunohistochemical stains, and molecular testing, allowing to identify the type and subtype of cancer, to assess the PD-L1 expression, and define the genomic profile. Staging tests may include imaging procedures that allow the clinician to search for evidence that the cancer has spread beyond the lungs. These tests include CT, magnetic imaging resonance (MRI), positron emission tomography (PET), and bone scans. The characterization and staging of the cancer will help define a treatment plan, combining one or more types: surgery, radiotherapy, and drug therapy (chemo- and immunotherapy); as well as the order that they are applied, taking into account the specific conditions of the patient. In cases where there is progress of the cancer after the line of treatment, the process of diagnosis will restart, using an imaging analysis, and if the tumor is so different from the expected, a biopsy will be performed.

### 2.3. Treatment Plan

Depending on the staging of lung cancer, patients are eligible for treatments that may be local, such as surgery and radiation therapy; systemic, such as chemotherapy and targeted therapy; or combined, meaning the merging of two or more types of treatments [38]. Early-stage NSCLC patients can be treated surgically with a 5-year-survival rate of 77% [39]. If stage I–II patients are unable to tolerate surgery (due, for example, to other associated health problems or inaccessible tumor location), they usually receive stereotactic body radiation therapy. If the tumor is found to be resectable from imaging studies and biopsies and the patient is able to tolerate surgery, surgery is typically performed to remove the tumor. However, most cases are identified in the late stages, when surgery is no longer an option as a result of distant metastases. Treatment for stage III NSCLC patients include some combination of radiation therapy and chemotherapy. Immunotherapy is a target therapy that attacks immune checkpoint pathways, which includes the blockade of the inhibitory receptors cytotoxic T-lymphocyte-associated antigen 4 (CTLA-4) and programmed cell death-1 (PD-1), and its ligand, PD-L1, and has altered the management of NSCLC over the last 10 years [40]. For stage IV NSCLC patients, who constitute 57% of newly diagnosed lung cancer patients [41], there are several lines of treatment, depending on whether the cancer cells have certain genetic or protein alterations, and the overall health of the patient. Personalized medicine by targeting appropriate genomic biomarkers with small-molecule tyrosine kinase inhibitors (TKIs) has helped improve survival in stage IV NSCLC patients, while decreasing multiple undesirable side effects associated with cancer treatment [42,43,44,45]. On lung cancer, one of the most relevant oncogenes and a predictive biomarker with clinically approved therapies is *EGFR* [46]. *EGFR*-dedicated therapies with TKIs, such as *afatinib* and *erlotinib*, are currently used as first- and second-line lung cancer treatments [47], improving objective response rates and progression-free survival compared to cytotoxic therapy for patients with mutated *EGFR* [48,49,50]. Furthermore, for patients whose tumor expresses the PDL-1 protein on at least 50% of the cells, the treatment options might include the administration of an immunotherapy drug, such as pembrolizumab, which is a human immune checkpoint inhibitor that can inhibit the PD-1 or PD-L1 and improve antitumor immunity [51,52,53]. However, immunotherapy is only effective for a small percentage of cancer patients (20%), due to the low performance of the current predictive biomarkers of the response to the immune checkpoint blockade therapy, which relies on the detection of PD- L1 in cancer tissue [54]. On the other hand, if the PD-L1 levels are lower than 50%, the treatment often consists of chemo- and immunotherapy combination.

### 2.4. Main Biomarkers for Target Therapies

Only a small portion of NSCLC patients are diagnosed at an early stage (I or II), when surgical resection is an optimal treatment option [55,56]. Researchers are focusing on developing targeted therapies because of the ability to deliver drugs effectively with high specificity while being less toxic. Target therapies identify and block specific enzymes, proteins, or other molecules involved in cancer development. Thus, understanding the pathophysiology of cancer is a crucial step to identify molecular targets that favor the promotion of cancer cell growth, interfere with the regulation of cell cycle, and/or induce cell death, so as to interfere within the tumor microenvironment and activate the immune system [57]. In NSCLC, one-third of the patients have an oncogenic driver mutation that is druggable, another third show excessive inflammation in the tumor micro-environment that can be targeted with an immune checkpoint, and the last third of patients are treated with combined chemotherapy [58]. In NSCLC, some specific targets that have already been studied are oncogenes, such as *EGFR*, *KRAS*, or *ALK*, and immune checkpoints, such as PD-1/PD-L1 and CTLA-4.

#### 2.4.1. Oncogenes

*EGFR* is a receptor found on the surface of cells and is a member of the *EGFR*-family of extracellular protein ligands that cannot penetrate the cell membrane; thus, they function via targeted signal transduction pathways that carry cellular information [59,60]. Ultimately, its function as a cell proliferation, differentiation, motility, and survival factor allows cancer cell growth and development, as well its metastasis [59]. It is a tyrosine kinase receptor that is frequently overexpressed in tumors, and so it is considered a predictor of survival [59]. The *EGFR* mutation occurs in 10–20% of patients with lung cancer (80–85% of NSCLC) and is mostly adenocarcinoma in younger women and never-smokers [56]. Within the *EGFR* mutation, the most common are the exon 19 deletion and exon 21 L858R point mutation—almost 90% of them—and they are also the ones with the better response to *EGFR*-targeted therapies [58]. For *EGFR*, there are two types of drugs: (1) monoclonal antibodies, which bind to the extracellular domain of *EGFR*, preventing its dimerization; (2) tyrosine kinase inhibitors, which block the intracellular part of the receptor [59,61].

The rat sarcoma proto-oncogene (*RAS*) family mutations are the most frequent cause for cancer, including three different oncogenes: the Kirsten rat sarcoma oncogene *KRAS*, the neuroblastoma rat sarcoma oncogene (*NRAS*) and the Harvey rat sarcoma oncogene (H-RAS) [62]. The *KRAS* isoform expresses the most alterations, accounting for 86% of RAS mutations, mainly in lung, pancreatic, and colon cancers [62,63,64]. *KRAS* mutations are responsible for about 30% of lung adenocarcinomas, with higher prevalence in Western countries and in smoking patients, showing worse outcomes in early and advanced stages of lung cancer [62,63,64].

*KRAS* is a guanosine triphosphate protein (GTPase) encoded by the *KRAS* oncogene and activated by cell surface receptors, such as *EGFR*, fibroblast growth factor receptor (*FGFR*), and human epidermal growth factor receptors 2–4 (*HER2-4*), which through downstream pathways will induce cell proliferation, differentiation, or cell death. Although the molecular processes involving the *RAS* family are quite known, there is no potent anti-RAS therapy as yet, since, for the last four decades, every targeted therapy to this molecular pathway has not shown good clinical results [62,63,64,65]. Reasons for that likely include heterogeneity involving the *RAS* mutations, mainly the ones in the *KRAS* oncogene, besides the co-occurring genetic events and the diverse *KRAS* allelic content that contributes to direct clinical implications [62].

*ALK* is a member of the transmembrane insulin receptor superfamily of receptor tyrosine kinases [59,66]. Although its mutations have been known for more than 10 years, the *ALK* role, as well as ligands, are still being debated [66]. In NSCLC, *ALK* mutations represent 2–7% mostly in never- to light-smokers, men, with 50 being the median age of diagnosis [56]. *ALK* tyrosine kinase inhibitors are ATP-competitive antagonists, preventing *ALK* kinase activity and promoting tumor reduction [59]. Crizotinib is an example of an *ALK* inhibitor, which reduces 50–60% of tumor sizes in patients with this mutation and provides greater improvement in one’s quality of life, although most of the patients had previous chemotherapy [59,66].

#### 2.4.2. Immunobiomarkers

**Figure 2:**
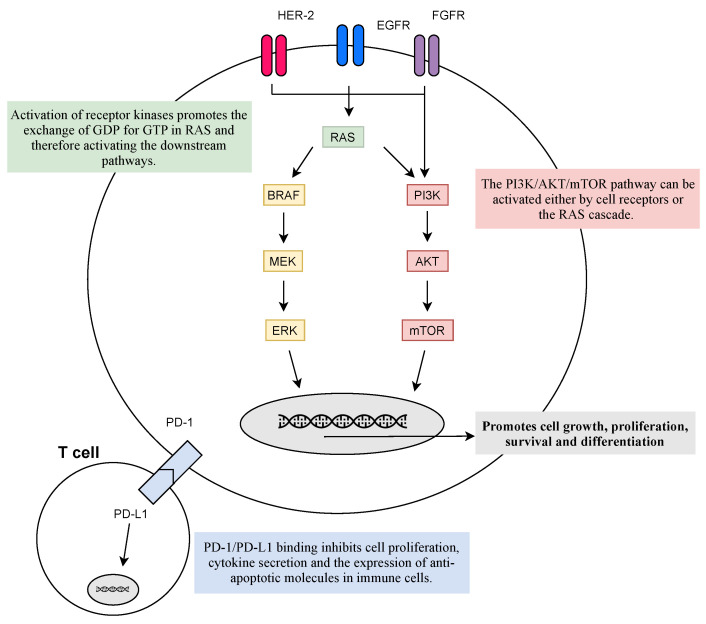
Physiological response to the activation of membrane receptors and immune receptors. Activation of receptor kinases, such as *EGFR*, *FGFR*, or *HER-2*, promotes the activation of *RAS* and its downstream pathways that facilitate cell growth, proliferation, cell survival, and differentiation. However, mutations in the *RAS* family lead to its constitutive activation and the hyperactivation of the downstream pathways—leading to uncontrolled cell survival. On the other hand, PD-L1 is present on the cell surface of immune cells and its binding to tumor cells PD-1 inhibits cell proliferation, cytokine secretion, and the expression of anti-apoptotic molecules in immune cells culminating in the escape of cancer from immunosurveillance. The goal of target therapies is to diminish the activation of abnormal signalling pathways, which can be inhibited at every step.

Inhibitory checkpoint molecules generated upon T cell activation, such as those that regulate the immunological synapses between T cells and dendritic cells in lymph nodes (CTLA-4 and B7.1), or between T cells and tumor cells (PD-1 and PDL-1/2), are currently the most relevant targets for immunotherapy (see Figure 2) [56].

PD-1 presents on the cell surface as a co-inhibitory receptor, expressed in T cells, B-cells, monocytes, and natural killer T cells after activation. Binding of PD-L1 to PD-1 inhibits cell proliferation, cytokine secretion, and the expression of anti-apoptotic molecules in immune cells [55,59,67]. In various malignancies, including lung cancer, PD-L1 is overexpressed, allowing the activation of PD-1 signalling pathways and ultimately the escape of cancer from immunosurveillance [67]. Blockage of PD-1/PD-L1 pathways has been the most successful strategy as it promotes the programmed death of tumor cells, with various anti-PD-1/PD-L1 antibodies approved for first- and second-line settings with manageable toxicity profiles, improved efficacy, and longer durations of response compared to standard chemotherapy [59,67,68].

CTLA-4 (or CD152) is a known receptor of an immune checkpoint pathway that downregulates T cell proliferation, mainly in lymph nodes, and promotes immune self-tolerance [59,67]. CTLA-4 is often overexpressed in a chronic inflammatory status, such as in cancer, implying that its presence in the tumor microenvironment may be involved in the dysregulation of the immune response [67]. Therefore, targeting CTLA-4 allows enhancing T cell-mediated anti-tumor activity [59,67]. Monoclonal antibodies, such as ipilimumab, prevent CTLA-4 binding to its ligands (CD80/CD86) and, thus, augmenting T cell activation [59,68].

## 3. Computer-Aided Decision Systems

Computer-aided decision systems could be defined as tools that automatically extract valuable information from medical data and help make more accurate and fast decisions. In lung cancer, CADs focus on using imaging results from CT scans and producing predictions that help the clinicians to decide the follow-up of the patients or the best treatment plans. An effective CAD should comprise various components: pre-processing, segmentation, feature extraction, classification, grading, and characterization of cancer. An ideal CAD would present specific features (e.g., accurate, non-invasive, low-cost, repeatable, generalizable, and interpretable) to be integrated into the clinical routine of the cancer assessment (see Box 1).

Box 1 **Key features of CADs for lung cancer diagnosis.**

**Accurate**: to be used to help clinicians produce better decisions;

**Non-invasive**: to avoid the problems associated with an invasive procedure;

**Low-cost**: to be implemented on a large scale;

**Repeatable**: allowing it to be performed several times to follow the progress and treatment results;

**Generalizable**: to be able to deal with the heterogeneities of the population and make correct predictions for unseen data;

**Interpretable**: to give additional information to clinicians to trust in the decision.

**Figure 3:**
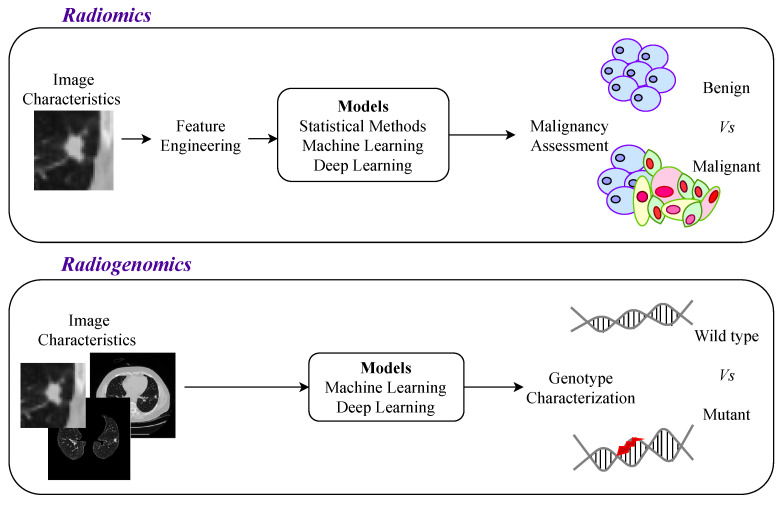
Radiomic vs. radiogenomic perspectives for lung cancer assessment. The assessment of lung cancer was first based on the nodule; however, recently, in radiogenomics approaches, other lung structures were shown to have relevant information for cancer characterization. Those approaches brought new challenges, such as the segmentation of lungs, to use this region of interest (ROI) in the AI-based models.

The developed approaches were first based on statistical methods (and more recently on machine learning models). The initial automatic methods attempted to correlate imaging features with the malignancy, which is defined as radiomics. The use of the most powerful methods opened up the possibility of exploring a characterization of cancer, such as the genotype (see Figure 3). Radiogenomics is an approach to predict the genotype (genes mutation status) based on imaging information (phenotype), which could reduce the need for biopsies.

For the particular case of lung cancer, there are specific elements that are crucial to consider in the phenotype characterization. The first element is the nodule, which is a cluster of tumor cells. This structure must be detected, segmented, and assessed to make the initial diagnosis of malignancy. For the malignant cases, CADs could help in cancer characterization, based on more information from the lung structures surrounding the nodules since other lung pathologies are correlated with cancer development [69].

Exploratory studies that have taken into account features from multiple lung structures, and did not just focus on the nodule, showed the importance of including extra-tumor features to obtain a successful genomic prediction (see Figure 3, where it is illustrated that radiogenomics approaches use information from more than just the nodule region) [50,69,70,71,72,73]. This seems to indicate that cancer development is related to multiple physiological changes not restricted to the nodule region and that the next generation of CADs should consider large lung regions to allow for a more complete lung cancer characterization [16,74]. This comprehensive approach, in the treatment planning field, would allow for the selection of a personalized treatment that would improve effectiveness and efficiency while diminishing avoidable therapy-related adverse events. This strategy may be particularly helpful in elderly or unfit patients who are at higher risk of procedure-related complications.

**Figure 4:**
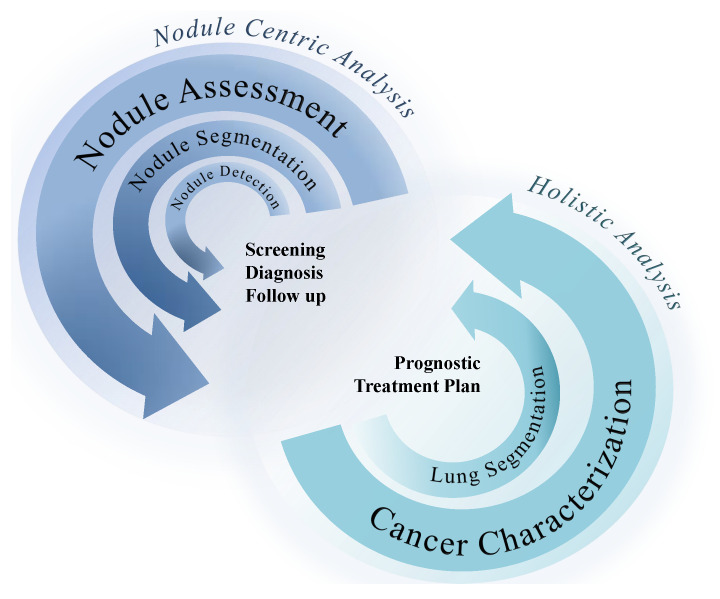
Two main perspectives for CADs in lung cancer, focused on the nodule and a more holistic approach that takes into consideration information about the surrounding structures of the nodule.

A deep characterization of lung cancer shows the need for more comprehensive analyses, capturing more information of other lung structures related to cancer development, which potentially present relevant information for more accurate predictions of the main biomarkers. Figure 4 illustrates the two main perspectives for AI-based CAD development for lung cancer, focusing on the nodule region or approaching a more holistic perspective of the lung condition. The following sections are dedicated toward analyzing the methodologies developed for nodule detection, segmentation, and classification; lung segmentation, genotype prediction, and other biomarkers prediction reflecting the movement from approaches centered in the nodule to more inclusive approaches. The selected works were presented in chronological order, with a deep discussion of the current limitations and possible opportunities and solutions. There are several up-to-date and significant reviews on each specific part of the CAD development solutions. The correspondent review papers are presented at the beginning of each section dedicated to CADs. The present work is dedicated toward capturing a global perspective of the general pipeline of CADs based on CT images dedicated to lung cancer evaluation; we present the specific challenges of each part of the clinical pathway and provide a critical discussion of the results.

### 3.1. Nodule-Focused CADs

This section is dedicated to the CAD centered in the nodule. This represents the first approach dedicated to lung cancer, following the clinical proceedings, by only taking into consideration the imaging findings presented by the nodule for the assessment. For this reason, CADs for nodule detections, assessments, and classifications were developed for cancer prediction in order to help the clinicians.

#### 3.1.1. Nodule Detection and Segmentation

The automated detection and segmentation of lung nodules is of great significance in the treatment of lung cancer and in increasing patient survival [75]. In clinical settings, radiologists must extract suspicious lung nodules from numerous images, a rigorous task since many destabilising factors, such as distraction and fatigue, as well as the limitations of professional experience, can contribute to misinterpretation of the available data. Therefore, several studies have focused on overcoming these difficulties, helping radiologists make more accurate diagnoses by proposing CAD systems that perform automatic detection and segmentation of lung nodules.

The articles presented in this section were retrieved from the following databases: Science Direct, IEEE Xplore, Web of Science, and PubMed. The most relevant keywords used during our searches were “lung”, “nodule”, “detection”, “segmentation”, “pulmonary”, “tumor”, “cancer”, “CAD”, and “CADe”, with various combinations of logical expressions containing “AND” and “OR”. Initially, we selected 134 articles. Then, we filtered them according to their relevance to the subject and presence/absence in previous reviews. This gave us 32 articles through December 2021. All of the pre-selected articles are from the current year or last year.

To acknowledge the most recent articles published in the field of CAD systems for lung nodule detection and segmentation, we highlight in this section two review articles from 2020 and 2021. Halder et al. [76] presented a systematic review of state-of-the-art approaches and their progress towards lung nodule detection in chest CT images. This review covered the published works from 2009 to April 2018. In the review by Gu et al. [77], AI-based algorithms and their applications in lung nodule detection, segmentation, and classification were reviewed. The aim of this review was to help better understand the performances of current approaches, limitations, and future trends in lung nodule analyses. This review article covered papers published up until December 2020.

##### Nodule Detection

The heterogeneity and high variability of nodule imaging characteristics bring significant complexity into this task, and so lung nodule detection can naturally be seen separated in two sub-modules: (1) where multiple candidates are first proposed, and (2) the nodule/non-nodule distinction is refined. Considering DL-based approaches, encoder–decoder architectures are widely used as the base methods for initial nodule detection [78,79,80,81,82,83,84,85]. The extraction of hand-crafted statistical, shape, and texture features also brought valuable information for candidate detection, being further classified by SVM [86,87] or by using ensemble strategies to combine the learning abilities of different classifiers [88]. Other traditional vision algorithms found successful results in juxtapleural nodules detection [89]. In the context of this problem, missing a true nodule should be more penalized than predicting too many false suspicions; however, there is an obvious effort in the literature to decrease false positive mistakes, mostly approached by combining different classification networks [78,90], using multi-scaled patches for capturing features at different expression levels [80,81,91,92], employing other classification algorithms, such as SVM [82,86,87,93,94,95], Bayesian networks, and neuro-fuzzy classifiers [95], or proposing a graph-based image representation with deep point cloud models [96].

On the other hand, single-stage methods have also been explored. In the work by Harsono et al. [97], inspired by RetinaNet [98] successes, transfer learning techniques were employed to make use of ImageNet [99] pre-trained architectures, building a modified feature pyramid network (FPN) to combine the feature maps obtained at specific dimension levels, outperforming the previous state-of-the-art. A different single-stage approach can be found in [100], where the YOLO v3 architecture was firstly adapted for lung nodule detection, showing capability of detecting these small imaging elements.

Table 1 summarizes the different nodule detection systems mentioned above in chronological order.

##### Nodule Segmentation

Although the popularity of deep learning approaches has caused a take over of the majority of nodule segmentation tasks, other learning algorithms have also been used. Machine learning-based approaches are still used for segmentation tasks. Hybrid models combining ML classifiers have been applied [101], standard level set image segmentations [102], or regions growing—that merge regions with similar features [103]. DL methods have shown capability of outperforming the results presented by the previous works. The U-Net, 3D-UNet, VNet approaches are the most common architectures applied [104,105,106]. The deep deconvolutional residual network was proposed for nodule segmentation, using a summation-based long skip connection from convolutional to deconvolutional parts of the network [107].

All of these methodologies are summarized in Table 2 in chronological order.

#### 3.1.2. Nodule Classification

Identification of lung nodule malignancy at the early stage has a positive impact on lung cancer prognosis. Therefore, there is a need for CAD systems to classify the lung nodule into benign and malignant types with maximum accuracy to avoid delays in diagnosis. This section of the review provides an overview of the current technology for lung nodule classification, a subject of study that is heavily explored by researchers who see the mortality rate increasing each day.

Science Direct, IEEE Xplore, Web of Science, and PubMed were the databases used during the search for articles pertaining to the classification of pulmonary nodules. The keywords used were “lung”, “nodule”, “classification”, “malignant”, “benign”, “pulmonary”, “tumor”, “cancer”, “CAD”, and “CADe”, with various combinations of logical expressions containing “AND” and “OR.” The articles were filtered according to their relevance, performance results, year of publication, and presence/absence in other reviews. Of the 33 articles selected, one was published in 2021 and the rest in 2020.

In recent years, many deep learning techniques have been used in lung nodule classification and have shown promising results when compared to other state-of-the-art machine learning methods. Thus, not surprisingly, the most recent review article found, published in the year 2021, and written by Naik and Edla, focused on 108 research papers, published up until 2019, which proposed novel deep learning methodologies for the nodule classification in lung CT scans [108].

The development of CAD systems for lung nodule malignancy has focused on a binary analysis, which basically resumes into finding imaging characteristics with values for distinguishing benign from malignant nodules. Although more complexity could be extracted from this problem (e.g., the assessment of more detailed malignancy levels), the literature still focused on the “two class” version of this problem.

Regarding nodule feature extraction, CNN has became the standard approach in this field, either with single-network approaches [109,110,111,112,113,114,115,116,117,118,119] or using ensemble strategies to combine multiple models [120,121,122,123,124,125]. A combination of local and more nodule-specific features with more global information is captured by processing the input image at different dimensions [126,127,128,129,130,131], enabling to bring together features from different levels of analysis. Regarding training techniques, the possibility of making use of ImageNet [99] pre-trained architectures, as in [111,113,132,133], was shown to provide improvements in the predictive ability. In works that explored multi-task learning strategies, taking advantage of related tasks to enhance the extraction of relevant information, features captured by generative models while discriminating real and fake lung nodules, have also shown valuable roles in training options [134,135], as well as the use of knowledge obtained by learning to reconstruct nodule images [120,124,136].

Although the majority of the literature on this topic relies on end-to-end neural network-based methodologies, algorithms, such as SVM, XGBoost, and KNN, have also been employed, serving as classifiers for previous extracted deep features [117,120], combined multimodal features [112], or hand-crafted features, such as nodule textures, intensity, and shape [137].

Table 3 summarizes the detailed nodule classification works with reported best performance in chronological order.

#### 3.1.3. Interpretability Methods for Nodule-Focused CADs

As mentioned in the previous section, nodule classification models based on deep learning (DL) algorithms are able to achieve the highest performances. However, DL models are considered the least interpretable machine learning models due to the inherent mathematical complexity; thus, not providing a reasoning for the prediction and, consequently, decreasing the trust in these models [138]. When utilizing these black-box models in the medical domain, it is critical to have systems that are trustworthy and reliable to the clinicians, therefore raising the need to make these approaches more transparent and understandable to humans [139].

Explainable AI (XAI) refers to techniques or methods that aim to find a connection between the input features and the prediction of the black-box; thus, looking to justify the decision and its reliability. Perceptive interpretability includes XAI methods that focus on generating interpretations that can be easily perceived by humans, despite not actually ‘unblackboxing’ the algorithm [140]. Visual explanations are the most commonly used XAI methodologies in deep learning image analysis approaches [141], namely in radiology image-based predictive models, where the trust on a CAD system can increase substantially by presenting the areas of a medical image with higher contribution to the prediction, along with the prediction itself [138].

A large portion of the most utilized XAI methods in the medical domain are *post-hoc* models, which consist of methods external to the already trained predictive model, performing evaluations on the predictions without altering the model itself. These are off-the-shelf agnostic methods that can be found in libraries, such as *PyTorch Captum* [142]. This post-model approach was implemented by Knapič et al. [139], where two popular *post-hoc* methods, local interpretable model-agnostic explanations (LIME), and SHAPley Additive exPlanations (SHAPs) were compared in terms of understandability for humans in the predictive model with the same medical image dataset.

Furthermore, in-model XAI methods for lung nodule classification were also implemented by Li et al. [143], where an importance estimation network returns a diagnostic visual interpretation that is utilized by the classifier for an irrelevant feature destruction process in each pooling layer. In the developed model, only the essential features are preserved in the visual interpretation, being the optimization of the model achieved by a trade-off between the accuracy of the model and the amount of information used in the classification. In Jiang et al. [144], a convolutional block attention module (CBAM) was implemented to develop a partially explainable classification model for pulmonary nodules, allowing to build a relationship between the features of the input images and the symptom descriptions and infer that the rationale of the network shows some correlation with the diagnosis of physicians.

The concern for interpretability is increasing, especially in the medical field, where there are higher stakes and responsibilities in the CAD systems that are implemented. However, the research in the area of interpretable models is still in progress, despite the recent rise in the development of this approach. The increase in research efforts of interpretable CAD systems is already noticeable, mainly regarding the verification and explanation of the predicted diagnosis, rather than the unravelling of the black-box [140]. These methods may show future potential, not only in providing trustworthy explanations to physicians, but also in assuring the reliability and consistency of the developed models.

#### 3.1.4. Discussion and Future Work: Nodule Detection, Segmentation, and Classification

In this review of current methods, direct comparisons of research results were hampered by the heterogeneity in the selection of included scans, different parameters for the algorithms, and inconsistent use of performance metrics and evaluation protocols. Overall, the selected works have shown good capabilities in the detection, segmentation, and classification of pulmonary nodules in CT images. We found that the machine learning techniques showed satisfactory performance results, while deep learning, especially CNN, outperformed conventional models and emerged as a promising approach. The main advantages of CNN lie in its ability to directly learn from a variety of data sources and automatically generate relevant and possibly unknown features, allowing for prompt and efficient development of CAD systems. The major challenge is achieving the robustness to diverse clinical data of varying quality. Although the availability of heterogeneous private datasets have shown to improve model performance, results comparability, and generalization become limited. Furthermore, to ensure robustness, the proposed methods need to be validated with sufficiently large datasets that include all nodule types and sizes. Thus, methods that were evaluated with fewer nodules will likely lose accuracy under clinical conditions where nodule types are more varied. The next challenge is the discrepancy or variability between the manual annotations. For image-based annotations, such as detection, segmentation, and classification, such variability may reflect a possible ceiling performance for AI-based methods. In addition, feature extraction serves as an important step in differentiating nodules from other anatomic structures present in lung lobes. Yet, the optimal set of features for nodule detection remain a subject of debate. Moreover, although deep-learning technologies avoid handcrafting and selecting image features, they instead require the selection of a loss function, network architecture, and an efficient optimization method, all of which influence the learning process. Additionally, the images used for training and testing of nodule analysis algorithms may have excluded pathological conditions in addition to lung nodule screening. Incorporating day-to-day chest CT images from multiple centers and dealing with these real-life situations are challenges and are reasons why manual correction and interaction are necessary to help physicians read the images.

##### Improvements Needed

To improve CADe and further develop its contribution to lung cancer treatment, some areas need to be explored:

1. Large and different public lung nodule databases for algorithm evaluation to provide replication of desired results and enhance the stringency of the algorithm so that lung nodule analysis tools can be validated mimicking real clinical scenarios.
2. The ability to deal with pulmonary nodules based on location (isolated, juxtapleural, or juxta-vascular) and internal texture (solid, semi-solid, ground-glass opacity, and non-solid). In particular, the detection of ground glass optical and non-nodules is difficult and is explored by very few researchers.
3. The ability to deal with pulmonary nodules with extremely small diameters. Most early-stage malignant tumors are smaller in size, and if these tumors are detected at an early stage, the survival chance of the individual can be increased.
4. The ability to classify nodules not only as benign or malignant, but as benign, early-stage cancerous nodule, primary malignant, and metastasis malignant, decreasing the level of abstraction related to some clinical phenomena that must be considered.
5. Develop a system capable of segmenting out large solid nodules attached to the pleural wall, which is quite challenging.
6. Build a set of useful and efficient features based mainly on shape or geometry, intensity, and texture for better false-positive reduction.
7. Develop a new CAD system based on powerful feature map visualization techniques to better analyze CNN’s decision and transfer it to radiologists.
8. Fine-tune a pre-trained CNN model instead of training it from scratch to increase its robustness and surpass the limitation of annotated medical data.
9. Develop in-depth research on GAN models, which can solve the problem of lack of medical databases.
10. Design new CAD systems, including two or more of the CNN architectures to address the problem of overfitting that occurs during the training process due to imbalance in the datasets.
11. Develop new deep learning techniques or optimize existing techniques to improve the performance of the CADe system, such as using a contracting path (to capture context) and a symmetric expanding path (to enable precise localization) to strengthen the use of available annotated samples, training multilayer networks efficiently by residual learning to gain accuracy from considerably increased depth.
12. Promote cooperation and communication between academic institutions and medical organizations to combine real clinical requirements and the latest scientific achievements.

### 3.2. Lung Segmentation

Lung segmentation is a critical task necessary in the majority of lung imaging CAD studies. Despite not being provided to radiologists in real scenarios, an accurate lung mask is absolutely crucial in the development of clinical support tools, avoiding the inclusion of noise and non-relevant background information, which also improves the efficiency of the computational resources usage. However, the main challenge to overcome remains the lack of robustness of the developed tools when analysing lung images with completely different properties. The large diversity of lung pathological status and biological phenomena associated with severe imaging manifestations often result in extremely difficult segmentation cases, and models tend to fail in these scenarios.

In the work by Shaziya et al. [145], a comprehensive review of the state-of-the-art solutions regarding conventional, machine learning, and deep learning solutions was made, collecting several works from 2001 to 2018. El-Baz et al. The authors of [146] also reviewed the most relevant challenges associated with the lung cancer diagnosis research field, including several works regarding the lung segmentation task. Since these were the only published review articles found on this subject, the works included in this section were carefully compared to ensure the absence of overlapping. The search queries selected were (“Lung segmentation”) AND (“CT”) using the IEEE Xplore, Science Direct, and PubMed databases, which resulted in a total of 26 selected articles. This section is divided into conventional and learning methods. The first includes a wide group of fundamental computer vision-based methodologies from 2014 to 2021. The second comprises a selection of machine and deep learning solutions from 2019 to 2021, considering the large amount of recent approaches and the articles already discussed in [145]. To facilitate the article search, the keyword “Nodule” was excluded from the title search option, excluding the amount of works only dedicated to nodule analysis.

#### 3.2.1. Conventional Methods

Approaching lung segmentation through conventional computer vision methods often requires manual interventions for initialization of the algorithm [147]. Filtering operations, such as histogram-based thresholding [148,149], may be susceptible to several abnormalities present in lung tissues with higher or lower density values compared with the rest of the lung. To overcome this, a possible direction was proposed by Shi et al. [149], consisting of combining the “weak” and the “strong” from the multiple methods, with the intuition that it would result in an improved segmentation ability over single-method approaches. Morphological operations were also used as a post-processing option to fine-tune the predicted masks by eliminating some common mistakes, such as holes inside the lung tissues [148].

More complex methodologies based on active contour models [150,151], and modifications on the random walker method [152] were also recently proposed. These methodologies showed a robustness increase, even in the presence of tissue abnormalities, also enabling more automatic pipelines at the same time. A multi-atlas segmentation approach for thoracic organs at risk (OAR) was also proposed by Oliveira et al. [153], by considering the spatial relationships between the different thoracic organs to produce a single spatially coherent mask.

Table 4 summarizes the reviewed conventional methodologies for lung segmentation in chronological order.

#### 3.2.2. Learning Methods

The most recent approaches for CT lung segmentation show a clear predominance of learning algorithms capable of directly learning the distribution of the data used for training. Methodologies inspired on U-net [154] cover the majority of deep learning-based attempts [155,156,157,158,159,160,161,162,163,164,165]. To increase the complexity of the feature extraction task, the encoder module could reuse transferred weights from pre-trained networks, as in the works by Vu et al. [163] and Jalali et al. [166], where the VGG-16 and ResNet-34 models were adopted to work as encoder blocks, respectively. More investigations on improvements in typical convolutional blocks can also be found, integrating residual blocks [164,167], inception modules with dense connections [162], and squeeze-and-excitation blocks to target specific thoracic organs at risk [165]. Still, on feature extraction enhancement, adversarial training approaches were explored in [155,168,169], enabling approximating the predicted masks to the ground-truth by discriminating between both. More meaningful features can also be extracted by aggregating an auxiliary classification branch, enriching the information used for backpropagation [160,170]. Liu et al. [171] integrated different feature extraction branches by combining deep, textured, and intensity features, to be classified as part of the lung mask or background.

In two-stage pipelines, approaches based on lung detection followed by proper segmentation of the cropped input have been proposed [156,172,173], the Mask R-CNN [174] architecture was employed and predictions were refined through combining different supervised and unsupervised methods. These regularisation techniques confirmed that, as expected, less noisy inputs would allow to obtain better predictions.

The lack of training data diversity has been recognized as a major barrier to achieve robust segmentation models, with better results obtained with larger and more heterogeneous private data, even with simple networks [158].

Table 5 summarizes the reviewed machine/deep learning methodologies for lung segmentation in chronological order.

##### Discussion and Future Work: Lung Segmentation

The use of conventional methods for lung segmentation has, to some extent, achieved satisfactory results for certain scenarios of data distributions. Image threshold-based algorithms often lack robustness, not being able to cope with higher variances on the density values of more heterogeneous lung structures. To achieve decent results, these algorithms require an extensive amount of post-processing work, employing highly data-dependent fine-tuning methods, which improves the performance by creating tight boundaries on the properties of a specific dataset. Regarding more dynamic algorithms, such as active contour models (ACM) and their variations, initial contours are often necessary for method initialization and the energy functions used for mask propagation can be susceptible to heterogeneous imaging variations in shape or intensity and, therefore, must be extensively tested over distinct sources of data to be considered clinically reliable.

The majority of the most recent segmentation approaches proposed incorporate deep learning mechanisms, allowing the development of completely automatic solutions without the need to design and apply specific algorithms to solve specific problems. Since the publication of U-net [154] as a general biomedical image segmentation network, multiple approaches have been proposed to improve segmentation capabilities by increasing the complexity of the network. To improve the knowledge obtained in the extracted feature maps, the inclusion of handcrafted imaging features and auxiliary guided classification branches are examples of some technical innovations that were proposed, motivated by the chance of increasing the information that could be used to deal with more heterogeneous tissue patterns.

However, there still exist several issues that have hindered the development of universal segmentation systems capable of being adopted in clinical routines. The differences in contouring guidelines between databases is a crucial discussion point when evaluating lung segmentation approaches. Several models are developed with ground-truth labels that may not be adequate for every context of analysis. In the LOLA11 data description section, the statement “… lung segmentation images are not intended to be used as the reference standard for any segmentation study.” alerted the authors for this issue when selecting the data sources for their segmentation experiments. In several databases with available lung masks, these were often obtained using automatic segmentation tools or previously developed algorithms. In the cases where the main purpose of the database publication was not related to the segmentation tasks, the criteria for the included patients were often biased for specific pathological diagnoses, which made it more difficult to obtain the desired diversity of patients. Moreover, since lung masks are made available as a supplement, the processes for the contour quality assurance, the agreement rates of the annotators, and the contouring guidelines are not disclosed in most cases. This problem is emphasized by the fact that discrepancies related to the inclusion or exclusion of certain regions, such as trachea, main/secondary bronchi, and tumor regions in training data may create a substantial impact on the quantitative evaluation and performance comparison of different segmentation models. From the articles reviewed, it is possible to see that, in general, the models developed using privately collected data achieved higher generalization abilities in comparison with the ones trained using only public data sources. However, the generalization achieved was still limited, caused by using data from one single healthcare institution or a single country, which created a significant bias on the data collected.

Considering these facts, universal segmentation tools are needed for the future where CAD systems are implemented in the clinical routines. Innovation on the modeling fundamentals should continue to be investigated, to increase generalization, in order to cope with the large heterogeneity of tissues caused by the pathological phenomena occurring in the lung structures. Moreover, the implementation of measures to encourage the sharing of biomedical data for research purposes would automatically push the challenges that researchers face while addressing such tasks, which would cause a massive improvement in the utility of their outcomes for the clinical practice.

### 3.3. Genotype Prediction

Genotype studies are the fundamental keys in the development of personalized medicine in lung cancer and they enable the progress of targeted therapies. Furthermore, gene analysis allows to identify biomarkers that can be used for early cancer detection, predict the prognosis and the response to the treatment plans, and monitor disease progression [175]. The most recent ambition for CAD has been to correlate the phenotype captured by the radiological images and determine the associated genotype. Recent studies have focused on predicting the *EGFR* mutation status using CT imaging, since targeted therapies for this gene already exist.

In total, twenty studies were found after employing the query (“Gene Mutation Status”) AND (“Prediction”) AND (“Lung Cancer”) in the research databases IEEE Xplore and PubMed, and excluding the ones that were not based on CT scans. These studies included semantic, radiomic, and deep features, which were the inputd of statistical, machine learning, or deep learning models. All of these studies were from 2017 to 2021, which shows how novel the investigation of this area is.

#### 3.3.1. Centered on Nodule

Thus far, twelve studies were found, dedicated to study gene mutation status prediction by CT scan analysis taking into account features related to the nodule. Table 6 provides detailed information of each work dedicated to genotype prediction using nodule features.

*EGFR* is the most relevant oncogene due to the frequency of occurrence and the target therapies available for clinical use. For these reasons, several CADs have been developed for the detection of the mutation status of this gene. Correlations between CT morphological features and the presence of *EGFR* mutations were studied and showed that the *EGFR* mutation tended to exist in tumors with part-solid GGO [176]. Approaches based on ML methods were extensively used and showed promising results [177,178]. A different approach was used to predict *EGFR* mutation status and to extract high-level deep features [179,180,181,182]; a CNN showed the best classification performance with an AUC of 0.85. Few other works were dedicated to identify the mutation statuses of other oncogenes, including *KRAS* [183,184], *ALK* [185] or even other genes (ERBB2 receptor tyrosine kinase 2 (*ERBB2*) and tumor protein 53 (*TP53*)) [186]. Those predictions were performed using ML-based approaches and considering radiomic features [183,184,185,186]. The best performance results obtained achieved AUC of 0.81 for *KRAS*, 0.87 for *ERBB2* and 0.84 for *TP53* [186].

#### 3.3.2. More Comprehensive Approaches

Thus far, seven studies were found that took into account at least one feature related to the structure or disease external to the nodule. Table 7 presents an overview of each work that used a more comprehensive approach for genotype prediction.

A comprehensive approach is based on the combination of information from nodule features, other lung structures, and a possible fusion with clinical data. The use of all this knowledge allows a deep characterization of the pathophysiological changes that occurred, which could benefit the prediction of the mutational status of the oncogenes. Part of the models developed on a more comprehensive analysis employed semantic imaging data annotated by thoracic radiologists that captured extensive regions on the lung and patient conditions, instead of focusing only on the nodule region. These approaches were based on radiological qualitative features [71,188,189]. On the other hand, the features from the CT images can be objective and automatically extracted, such as radiomic or high-level deep features [190,191,192]. Additionally, both types of features (semantic features and the automatically extracted) can be used together by the learning models [50].

The studies that used semantic features combined with the simplest classification models allowed the assessment of the most relevant lung and nodule features for the mutation status prediction. The wild type status for *EGFR* was predicted by the appearance of emphysema and airway abnormality while the presence of any ground glass component indicates *EGFR* mutations [71]. Moreover, gender, smoking history, emphysema, diameter in the mediastinal, TDR, and GGO showed statistical differences between the wild type group and mutated group of *EGFR* [188]. The connection between *EGFR* mutation and internal air bronchogram, pleural retraction, emphysema, and lack of smoking was found [189]. The mixing nodule-related features with features from other lung structures showed to benefit the *EGFR* mutational status prediction [50].

The *KRAS* mutation status prediction showed non-consensual results even in these more comprehensive studies, and in some studies, this oncogene status was not connected with image features [50,71].

#### 3.3.3. Discussion and Future Work: Genotype Prediction

Radiogenomic approaches used to classify the mutation status of oncogenes for lung cancer patients have shown that there are radiomic signatures in CT images that can be used to distinguish mutant from wild type statuses. Previous studies have also demonstrated that radiological features, corresponding to descriptive features more familiar for radiologists, may be associated with tumor biology. Subsequent studies further demonstrated that the combination of radiomic features and the inclusion of clinical information strengthens the robustness of predictive models. Furthermore, recent studies that have taken into consideration features from a larger region of analysis that contained other structures from the lung appear to have more accurate predictive performances compared to traditional nodule-based approaches. Since lung cancer development is related to multiple physiological changes not restricted to the nodule region, it is expected that the studies that employ comprehensive approaches and consider extra-tumor features from the lung with the tumor obtain a significant increase in predictive performance. It is crucial to highlight these results and further investigate the importance of holistic lung cancer characterization studies, as many complex combinations of morphological, molecular, and genetic alterations occur during lung cancer development that, when taken into account, would allow the development of more accurate predictive models.

The value of image analysis to reveal biological information will not completely replace the need for tissue biopsy or liquid biopsy. However, image-driven studies can provide additional information that is complementary to biopsies. For example, if the biopsy result of a tumor shows *EGFR*-wild type, the result may include false negatives because of intra-tumor heterogeneity. At this time, the learning model can be seen as an alternative validation tool, as CT imaging provides biological information that can describe the genotype and phenotype of the whole tumor and project the biological information onto each pixel of images to reflect intra-tumor heterogeneity. If it predicts the tumor to be *EGFR*-mutant, clinicians may need to re-biopsy tissues. In addition, predicting mutation status by CT imaging helps us to choose the most suspicious tumor for biopsy if multiple tumors are present in a patient. Finally, the predictive model requires only routinely used CT imaging, which is a non-invasive technique and easy to acquire throughout the course of treatment. The CT scan can be performed multiple times along the treatment plan, allowing to assess the treatment response of the patient. Multiple assessment throughout the treatment plan may not be possible to perform by biopsy due to its invasive nature. Therefore, it is worthwhile to develop an image analysis to complement the tissue biopsy and liquid biopsy for more precise systemic treatment and local therapy.

The radiogenomics field presents a small number of publications that are strongly limited by the small sizes of the available databases, which are hardly a good representation of the population affected by lung cancer. In addition, there is a larger number of benign nodules compared to malignant ones in the available public databases, which hinder the ability to extract useful features related to malignant cases only. Furthermore, performance comparisons between models trained and tested with different data do not allow clear and objective conclusions, and image acquisition protocols and performance validation methods (i.e., cross-validation) differ from study to study. Still, direct quantitative comparisons on prediction results are crucial for a clearer understanding of the research evolution, increasing the need for a large and heterogeneous cohort of patients affected by lung cancer, as well as methods capable of coping with data heterogeneity. Accordingly, the sharing of image data among different clinical institutions, but under an uniform protocol to avoid any inconsistency during data record, is valuable to obtain an unique reliable dataset.

Before translation into clinical practice, multisite trials are also needed to validate the results obtained in training cohorts on separate independent groups of patients. Since a model fitting is optimal in the training set used to build the model itself, it is crucial to validate the model in a large external cohort of patients to obtain more reliable fitting estimates. External validation will determine the transportability of the model in different locations consisting of plausibly similar individuals.

Studying the variability amongst radiologists in multi-institutional cohorts is required in the near future to further study the robustness of the annotation of semantic features. Moreover, explainable AI is a field that should be further explored in radiogenomics studies, as it is important not only to consider black-box models, but also interpretable models whose predictive decisions can be understood by human observers.

### 3.4. PD-L1 Expression Prediction

The expression of programmed death-ligand 1 (PD-L1) can be used to predict the response of immunotherapy for lung cancer patients. Immunohistochemical (IHC) is the current method employed to detect PD-L1 expression levels, and its limitations related to the tumor heterogeneity have encouraged the development of more comprehensive and automatic approaches. In this section, a compilation of the most recent articles addressing the relationship between PD-L1 expression status and clinical imaging data are presented. Any published review article dedicated to this subject was found. A total of five articles were found after employing the query (“PD-L1”) AND (“Lung Cancer”) AND (“CT”) in the IEEE Xplore and PubMed databases. A summary of the key points regarding each included article is presented in Table 8.

Some of the studies dedicated to this biomarker used a statistical analysis to assess the relationship between PD-L1 expression and qualitative features extracted from CT scans, such as surrounding GGO, air bronchogram, and pleural indentation [193,194]. More powerful methods, such as DenseNet, showed improvements in assessing the PD-L1 expression status [195]. Tian et al. [196] studied the ability to use a combination of clinical, radiomic, and high-level deep features to predict the expression of PD-L1. However, ML methods using radiomic features extracted from the nodule showed the best performance results [197,198]. Yang et al. [199] developed a recurrent neural network (RNN) model and simple temporal attention (SimTA) modules that take into consideration asynchronous time-series imaging (radiomic features extracted from lung lesions) and laboratory data (PD-L1 expression and blood profile). Methods that allow the assessment will be very helpful in the understanding of the progress of disease and treatment response.

#### Discussion and Future Work: PD-L1 Expression Assessment

A new era of more precise treatment strategies for lung cancer patients emerged recently, such as the development of immune checkpoint inhibitors against PD-L1. The prediction of the expression status of PD-L1 it is intended to anticipate whether immunotherapy would be a successful treatment strategy or not. For this, the extraction of information from CT images has enabled the development of non-invasive and more comprehensive methods to predict the expression of PD-L1 in lung cancer patients.

However, the development of CAD systems capable of extracting quantitative information from CT images to assess the PD-L1 expression is a recent research field, and some issues have been identified as crucial barriers that limit the reliability of these methods. The lack of public databases has led to the dependence on privately collected data from clinical institutions, creating a bias on the obtained results of each individual study. Moreover, this issue makes comparisons over different works almost impossible, since single-center data do not present a rich diversity of cancer stages, histologic subtypes, and specific CT findings, which can induce variable correlations only related to the data under analysis. This challenge is transversal to recent imaging-related AI applications in the clinical field, and data sharing among multiple institutions must be considered as an urgent solution that will certainly improve the scientific evolution in these challenging tasks.

Considering the methodologies that have been designed to address this task, the bias induced by the lack of representative data have shown that simple correlation analyses among clinical, qualitative, or quantitative extracted features, and the expression of PD-L1, often result in distinct, and sometimes, contradictory conclusions over different studies. This issue emphasises the need to explore causality-based methodologies, which would enable understanding of asymmetric cause–effect relationships between the clinical and imaging characteristics of the patient and the outcome result. Developing models capable of capturing the causal relationships between data will increase their reliability and future applicability in clinical routines.

## 4. Discussion and Conclusions

Currently, a large amount of data are being used by clinicians for diagnoses, which come from medical images, medical records, lab test results, and genomic data, and more recently, from wearable, mobile phone data, and environment data [200]. All of these data could be helpful in understanding and characterizing pathological events. A holistic approach could help to produce knowledge of why and when a pathological process is triggered. However, to process this amount of data, and to find the correlations and causal relations, the most powerful AI-based methods will be needed. Additionally, data from the liquid biopsy could allow the finding of new biomarkers based on the circulating elements related to cancer development. Cancer microbiome analyses suggest alternative perspectives for predictive techniques [201]. Novel biomarkers will help to stratify the cancer patients and find the best treatment plans for each one [202]. There is a close and dependent relationship between the use of novel sources of data, knowledge of the elements involved in cancer development, and innovative predictive approaches to help in clinical decisions. The current review was focused on CADs based on imaging data, but the future of the predictive approaches should be based on multimodal analyses.

Another relevant feature of future approaches will be the transparency of the models in order to allow the clinicians to understand and trust the model’s decision. Current methods include black-boxes that do not provide an explanation for the prediction obtained. For the next generation of CADs, “more than good” performance on the prediction will be needed, as well as the reasons for the prediction, in order to verify that correct information is used by the model and to extract new knowledge from the interpretation of the explanations. The ability to find causal relationships between the extracted features and the predictions is a challenging problem and constitutes a key step toward explaining model decisions [203,204].

Lack of large datasets remains a problem for healthcare solutions since models cannot learn how to deal with complex data due to insufficient training samples [205,206]. The difficult access to the medical data has slowed the progress of CAD development. Some alternatives have been proposed, such as federated learning; however, this limitation is not completely solved.

CADs dedicated to lung cancer—even with the current limitations and open challenges discussed here—are shown to be of great help to clinicians, regarding the multiple decisions that comprise clinical routines in lung cancer, from the initial evaluation and diagnosis to the treatment plan. AI-based solutions contribute to a more accurate and quick diagnosis in the early stages, reducing human error, and decreasing the costs. AI applications in healthcare could create a new era in cancer management.

## Figures and Tables

**Table 1:** Overview of published works regarding nodule detection approaches in lung CT images (2020–2021).

Authors	Year	Dataset	Methods	Performance Results (%)
Tan et al. [78]	2020	LIDC-IDRI	3D CNNs, based on FCN, DenseNet, and U-Net	TPR = 97.5
Mukherjee et al. [88]	2020	LIDC-IDRI	Ensemble stacking	ACC = 99.5 TPR = 99.2 TNR = 98.8 FPR = 1.09 FNR = 0.85
Shi et al. [79]	2020	LUNA16	3D Res-I and U-Net network	TPR = 96.4 FROC = 83.7
Khehrah et al. [86]	2020	LIDC-IDRI	SVM	ACC = 92 TPR = 93.7 TNR = 91.2 PPV = 83.3 MCC = 83.8
Kuo et al. [87]	2020	LIDC-IDRI Private (320 patients)	SVM	TPR = 92.1
Zheng et al. [80]	2020	LIDC-IDRI	3D multiscale dense CNNs	TPR = 94.2 (1.0 FP/scan), 96.0 (2.0 FPs/image)
Paing et al. [89]	2020	LIDC-IDRI	Optimized random forest	ACC = 93.1 TPR = 94.9 TNR = 91.4
Liu et al. [100]	2020	LIDC-IDRI	CNN algorithm: You Only Look Once v3	TPR = 87.3
Harsono et al. [97]	2020	LIDC-IDRI Private (546 patients)	I3DR-Net	mAP = 49.6 (LIDC), 22.9 (private) AUC = 81.8 (LIDC), 70.4 (private)
Xu et al. [81]	2020	LUNA16	3D CNN networks: V-Net and multi-level contextual 3D CNNs	TPR = 93.1 (1.64 FP/scan) CPM = 75.7
Drokin and Ericheva [96]	2020	LIDC-IDRI	Algorithm for sampling points from a point cloud	FROC = 85.9
El-Regaily et al. [90]	2020	LIDC-IDRI	Multi-view CNN	ACC = 91.0 TPR = 96.0 TNR = 87.3 F-score = 78.7
Ye et al. [82]	2020	LUNA16	Three modified V-Nets with multilevel receptive fields	ACC = 66.7 TPR = 81.1 PPV = 78.1 F-score = 78.7
Baker and Ghadi [93]	2020	LIDC-IDRI	SVM	NRR = 94.5 FPR = 7 cluster/image
Halder et al. [94]	2020	LIDC-IDRI	SVM	ACC = 88.2 TPR = 86.9 TNR = 86.9
Jain et al. [83]	2020	LUNA16	SumNet	ACC = 94.1 TNR = 94.0 DSC = 93.0
Mahersia et al. [95]	2020	LIDC-IDRI	SVM, Bayesian back-propagation neuronal classifier and neuro-fuzzy classifier	NRR = 97.9 (neuronal classifier), 97.3 (SVM), 94.2 (neuro-fuzzy classifier)
Mittapalli and Thanikaiselvan [91]	2021	LUNA16	Multiscale CNN with Compound Fusions	CPM = 94.8
Vipparla et al. [92]	2021	LUNA16	3D Attention-based CNN architectures: MP-ACNN1, MP-ACNN2 and MP-ACNN3	CPM = 93.1
Luo et al. [84]	2021	LUNA16	SCPM-Net	TPR = 92.2 (1 FPs/image), 93.9 (2 FPs/image), 96.4 (8FPs/image)
Bhaskar and Ganashree [85]	2021	DSB-2017	Gaussian mixture convolutional auto encoder + 3D deep CNN	ACC = 74.0

ACC: accuracy; AUC: area under the ROC curve; CPM: competition performance metric; DSC: Sørensen–Dice coefficient; FDR: false discovery rate; FNR: false negative rate; FP: false positive; FPR: false positive rate; FROC: free-response receiver operating characteristic; mAP: mean average precision; MCC: Matthews correlation coefficient; NPV: negative predictive value; NRR: nodule recognition rate; PPV: positive predictive value; TNR: true negative rate; TPR: true positive rate.

**Table 2:** Overview of the published works regarding nodule segmentation approaches in lung CT images (2020–2021).

Authors	Year	Dataset	Methods	Performance Results (%)
Sharma et al. [101]	2020	SPIE-AAPM Lung CT Challenge	SVM + k-NN	ACC = 93.9 TPR = 94.5 GM = 94.2
Xiao et al. [104]	2020	LUNA16	3D-UNet + Res2Net Neural Network	TPR = 99.1 DSC = 95.3
Singadkar et al. [107]	2020	LIDC-IDRI	Deep deconvolutional residual network	DSC = 95.0 JI = 88.7
Kumar and Raman [105]	2020	LUNA16	V-Net (3D CNN)	DSC = 96.1
Rocha et al. [106]	2020	LIDC-IDRI	Sliding Band Filter + U-Net + SegU-Net	DSC = 66.3 (SBF), 83.0 (U-Net), 82.3 (SegU-Net)
Hancock and Magnan [102]	2021	LIDC-IDRI	Level set machine learning method	DSC = 83.6 JI = 71.8
Savic et al. [103]	2021	LIDC-IDRI Private—phantom (108 patients)	Algorithm based on the fast marching method	DSC = 93.3 (solid round nodules), 90.1 (solid irregular nodules), 79.9 (non-solid nodules), 61.4 (cavity nodules)

ACC: accuracy; DSC: Sørensen–Dice coefficient; GM: Geometric mean; JI: Jaccard index; TPR: true positive rate.

**Table 3:** Overview of published works regarding nodule classification approaches in lung CT images (2020–2021).

Authors	Year	Dataset	Methods	Performance Results (%)
Wang et al. [109]	2020	Private (1478 patients)	Adaptive-boost deep learning strategy with multiple 3D CNN-based weak classifiers	ACC = 73.4 TPR = 70.5 TNR = 76.2 PPV = 83.8 AUC = 82.0 F-score = 71.6
Xiao et al. [120]	2020	LIDC-IDRI	ResNet-18 + Denoising autoencoder classifier + handcrafted features	ACC = 93.1 TPR = 81.7 PPV = 83.8 AUC = 82.0
Wang et al. [127]	2020	LUNGx	ConvNet	ACC = 90.4 TPR = 88.7 TNR = 92.4 AUC = 94.8
Lin et al. [110]	2020	LUNA16	GVGG + ResCon network	TPR = 92.5 TNR = 96.8 PPV = 93.6 F-score = 93.0
Onishi et al. [134]	2020	Private (60 patients)	M-Scale 3D CNN	TPR = 90.9 TNR = 74.1
Zhao et al. [126]	2020	LIDC-IDRI	Multi-stream multi-task network	ACC = 93.9 TPR = 92.6 TNR = 96.2 AUC = 97.9
Zia et al. [132]	2020	LIDC-IDRI	Multi-deep model	ACC = 90.7 TPR = 90.7 TNR = 90.8
Jiang et al. [121]	2020	LUNA16	Ensemble of 3D Dual Path Networks	ACC = 90.2 TPR = 92.0 FPR = 11.1 F-score = 90.4
Bao et al. [131]	2020	LIDC-IDRI	Global-local residual network	ACC = 90.4 TPR = 90.1 PPV = 89.9 AUC = 96.1
Shah et al. [111]	2020	LUNA16	NoduleNet (transfer learning from VGG16 and VGG19 models)	ACC = 95.0 TPR = 84.0 TNR = 97.0
Tong et al. [112]	2020	LIDC-IDRI	3D-ResNet + SVM with RBF and polynomial kernels	ACC = 90.6 TPR = 87.5 TNR = 94.1
Xu et al. [128]	2020	LIDC-IDRI	Multi-scale cost-sensitive methods	ACC = 92.6 TPR = 85.6 TNR = 95.9 PPV = 90.4 AUC = 94.0 F-score = 87.9
Huang et al. [113]	2020	LIDC-IDRI	Deep transfer convolutional neural network + Extreme learning machine	ACC = 94.6 TPR = 93.7 TNR = 95.1 AUC = 94.9
Naik et al. [122]	2020	LUNA16	FractalNet + CNN	ACC = 94.1 TPR = 97.5 TNR = 86.8 AUC = 98.0
Zhang et al. [118]	2020	LUNA16	3D squeeze-and-excitation network and aggregated residual transformations	ACC = 91.7 AUC = 95.6
Liu et al. [123]	2020	LIDC-IDRI	Multi-model ensemble learning architecture based on 3D CNNs: VggNet, ResNet, and InceptionNet	ACC = 90.6 TPR = 83.7 TNR = 93.9 AUC = 93.0
Afshar et al. [129]	2020	LIDC-IDRI	3D Multi-scale Capsule Network	ACC = 93.1 TPR = 94.9 TNR = 90.0 AUC = 96.4
Lyu et al. [114]	2020	LIDC-IDRI	Multi-level cross ResNet	ACC = 92.2 TPR = 92.1 TNR = 91.5 AUC = 97.1
Wu et al. [115]	2020	LIDC-IDRI	Deep residual network (ResNet + residual learning + migration learning)	ACC = 98.2 TPR = 97.7 TNR = 98.3 PPV = 98.5 F-score = 98.1 FPR = 1.60
Lin and Li [116]	2020	LIDC-IDRI	Taguchi-based AlexNet CNN	ACC = 99.6
Kuang et al. [135]	2020	LIDC-IDRI	Combination of a multi-discriminator generative adversarial network and an encoder	ACC = 95.3 TPR = 94.1 TNR = 90.8 AUC = 94.3
Lima et al. [137]	2020	LIDC-IDRI	SVM with Gaussian kernel + Relief + Evolutionary Genetic Algorithm	AUC = 85.6
Veasey et al. [133]	2020	NLST	Recurrent neural network with 2D CNN	PPV = 55.9 (t0), 66.9 (t1) AUC = 80.6 (t0), 83.5 (t1)
Bansal et al. [117]	2020	LUNA16	Deep3DSCan	TPR = 87.1 TNR = 89.7 AUC = 88.3 F-score = 88.5
Zhai et al. [124]	2020	LUNA16 LIDC-IDRI	Multi-task learning CNN	TPR = 84.0 (LUNA16), 95.6 (LIDC-IDRI) TNR = 96.8 (LUNA16), 88.9 (LIDC-IDRI) AUC = 97.3 (LUNA16), 95.6 (LIDC-IDRI)
Paul et al. [125]	2020	NLST	Ensemble of CNNs	ACC = 90.3 AUC = 96.0 TPR = 73.0 FNR = 27.0
Ali et al. [119]	2020	LIDC-IDRI LUNGx	Transferable texture CNN	ACC = 96.6 (LIDC-IDRI), 90.9 (LUNGx) TPR = 96.1 (LIDC-IDRI), 91.4 (LUNGx) TNR = 97.4 (LIDC-IDRI), 90.5 (LUNGx) AUC = 99.1 (LIDC-IDRI), 94.1 (LUNGx)
Silva et al. [136]	2020	LIDC-IDRI	Transfer learning (convolutional autoencoder)	AUC = 93.6 PPV = 79.4 TPR = 84.8 F-score = 81.7
Xia et al. [130]	2021	LIDC-IDRI	Gradient boosting machine algorithm	ACC = 91.9 TPR = 91.3 F-score = 91.0 FPR = 8.00

ACC: accuracy; AUC: area under the ROC curve; FNR: false negative rate; FPR: false positive rate; PPV: positive predictive value; TNR: true negative rate; TPR: true positive rate.

**Table 4:** Overview of published works regarding conventional methodologies for the segmentation of lung CT images (2014–2021).

Authors	Year	Dataset	Methods	Performance Results (%)
Lai and Wei [148]	2014	Private (10 patients)	Filtering process + morphological operations (threshold, region filling, closing)	TPR = 97.0 TNR = 99.0 AAE = 1.58
Li et al. [147]	2015	Private (15 patients)	Edge-based recursive geometric active contour (GAC) model	OV = 98.0
Shi et al. [149]	2016	Private (23 patients)	Histogram thresholding + region growing and random walk	OR = 1.87 UR = 2.36 ABD = 0.620 mm
Zhang et al. [150]	2017	LIDC-IDRI	Region- and edge-based GAC (REGAC) method	DSC = 97.7 HD-95 = 2.50 mm
Rebouças Filho et al. [151]	2017	Private (40 patients)	3D ACACM	F-score = 99.2 (ACACM), 97.6 (RG), 97.4 (OsiriX), 97.2 (LSCPM)
Oliveira et al. [153]	2018	VISCERAL Anatomy3	Multi-atlas alignment + label fusion (voting and statistical selection)	DSC = 97.4 (LL), 97.9 (RL) HD-95 = 4.65 mm (LL), 2.81 mm (RL)
Chen et al. [152]	2021	LOLA11 Private (65 patients)	Random walker	(Private) DSC = 98.6 (LL), 98.5 (RL) (LOLA11) DSC = 97.4

AAE: average area error; ABD: absolute border distance; ACM: active contour method; DSC: Sørensen–Dice coefficient; LL: left lung; LSCPM: level-set based on coherent propagation method; HD: Hausdorff distance; OR: over-segmentation rate; OV: overlap volume; RG: region growing; RL: right lung; TPR: true positive rate; TNR: true negative rate; UR: under-segmentation rate.

**Table 5:** Overview of published works regarding learning-based methodologies for the segmentation of lung CT images (2019–2021).

Authors	Year	Dataset	Methods	Performance Results (%)
Dong et al. [155]	2019	LCTSC	U-net generator with a FCN discriminator	DSC = 97.0
Feng et al. [156]	2019	LCTSC	Two-stage segmentation process with 3D U-net	DSC = 97.2 (RL), 97.9 (LL)
Park et al. [157]	2019	LCTSC Private (30 patients)	U-net	DSC = 98.8 JSC = 97.7 MSD = 0.270 mm HSD = 25.5 mm
Hofmanninger et al. [158]	2020	LCTSC, LTRC, VISCERAL, VESSEL12 Private (5300 patients)	U-net, ResUNet, Dilated residual network-D-22, DeepLab v3+	(merged dataset) DSC = 98.0 HD95 = 3.14 mm MSD = 0.620 mm
Yoo et al. [159]	2020	HUG-ILD Private (203 patients)	2D and 3D U-net	(Private - 2D; 3D) DSC = 99.6; 99.4 TPR = 99.5; 99.1 PPV = 99.6; 99.7 HD = 17.7 px; 18.7 px (HUG-ILD - 2D; 3D) DSC = 98.4; 95.3 TPR = 98.7; 98.0 PPV = 98.1; 92.8 HD = 7.66 px; 15.6 px
Khanna et al. [167]	2020	LUNA16 VESSEL12 2HUG-ILD	ResUNet + false positive removal algorithm	(LUNA16) DSC = 96.6 JI = 93.4 TPR = 97.5 (VESSEL12) DSC = 98.3 JI = 97.9 TPR = 98.8 (HUG-ILD) DSC = 98.1 JI = 96.3 TPR = 98.3
Shi et al. [160]	2020	StructSeg 2019	TA-Net	DSC = 96.8 (LL), 97.1 (RL) HD = 0.188 mm (LL), 0.171 mm (RL)
Nemoto et al. [161]	2020	NSCLC-Radiomics	2D and 3D U-net	DSC = 99.0 (2D/3D U-net)
Zhang et al. [162]	2020	Lung dataset (Kaggle “Finding and Measuring Lungs in CT Data” competition)	Dense-Inception U-net (DIU-net)	DSC = 98.6 JI = 98.7 ACC = 99.4 TPR = 98.5 TNR = 99.8 F-score = 98.5 AUC = 99.0
Vu et al. [163]	2020	Private (168 patients)	U-net with pre-trained VGG16	DSC = 97.0 (RL and LL) HD-95 = 5.10 mm (RL), 4.09 mm (LL)
Liu et al. [171]	2020	HUG-ILD	Random forest fusion classification of deep, texture and intensity features	DSC = 96.4 JI = 91.1 OR = 5.04 UR = 4.76
Hu et al. [172]	2020	Private (39 patients)	Mask R-CNN + supervised and unsupervised classifiers	DSC = 97.3 ACC = 97.7 TPR = 96.6 TNR = 97.1
Han et al. [173]	2020	Private	Xception + VGG with SVM-RBF Detectron2 + contour fine-tuning	DSC = 97.0 ACC = 99.0 TPR = 96.5 TNR = 99.4
Xu et al. [170]	2021	Private (217 patients) COVID-19-CT-Seg HUG-ILD VESSEL12	Boundary-Guided Network (BG-Net)	DSC = 98.6 (Private), 96.5 (StructSeg), 98.9 (HUG-ILD), 99.5 (VESSEL12) HD = 2.77 mm (Private), 1.39 mm (StructSeg), 0.665 mm (HUD-ILD), 1.40 mm (VESSEL12)
Jalali et al. [166]	2021	LIDC-IDRI	ResBCDU-Net	DSC = 97.1
Wang et al. [164]	2021	Lung dataset (Kaggle “Finding and Measuring Lungs in CT Data” competition)	HDA-ResUNet	DSC = 97.9 JI = 96.0 ACC = 99.3
Tan et al. [168]	2021	LIDC-IDRI QIN lung CT dataset	LGAN	(LIDC-IDRI) IOU = 92.3 HD = 3.38 mm (QIN) IOU = 93.8 HD = 2.68 mm
Pawar and Talbar [169]	2021	HUG-ILD	LungSeg-Net	DSC = 96.3 (Fibrosis), 96.5 (Ground glass), 91.4 (Reticulation), 97.6 (Consolidation), 97.8 (Emphysema), 99.0 (Nodules) JI = 93.7 (Fibrosis), 93.9 (Ground glass), 86.9 (Reticulation), 95.3 (Consolidation), 96.2 (Emphysema), 98.0 (Nodules)
Cao et al. [165]	2021	StructSeg 2019	C-SE-ResUNet	DCS = 97.0 (LL) 96.6 (RL)

ACC: accuracy; AUC: area under the ROC curve; DSC: Sørensen–Dice coefficient; HD: Hausdorff distance; IOU: intersection over union; JI: Jaccard index; LL: left lung; OR: over-segmentation rate; PPV: positive predictive vale; RL: right lung; TNR: true negative rate; TPR: true positive rate; UR: under-segmentation rate.

**Table 6:** Overview of published studies regarding predictive models for gene mutation status based on nodule features (2017–2021).

Authors	Year	Dataset	Methods	Performance Results (%)
Zou et al. [177]	2017	Private (209 patients)	Multivariable analyses	*EGFR:* AUC = 73.7
Cheng et al. [176]	2017	Private (2146 patients)	Weighted mean difference, inverse variance	*EGFR:* OR = 49.0
Li et al. [179]	2018	Private (1010 patients)	Random forest/CNNs	*EGFR:* AUC = 83.4
Koyasu et al. [178]	2019	NSCLC-radiogenomics	XGBoost/random forest	*EGFR:* AUC = 65.9
Wang et al. [180]	2019	Private (844 patients)	CNNs	*EGFR:* AUC = 85.0
Zhao et al. [181]	2019	TCIA and private (879 patients)	3D DenseNet	*EGFR:* AUC = 75.8
Moreno et al. [183]	2021	NSCLC-radiogenomics	SCAV with ML/CNN	*EGFR:* AUC = 82.0 (CNN) *KRAS:* AUC = 73.9 (CNN)
Zhang et al. [182]	2021	Private (914 patients)	Machine learning (SVM/RF/MLP) Deep learning (SE-CNN/CNN/1D-CNN/AlexNet/Fine-tuned VG16/Fine-tuned VGG19)	*EGFR:* AUC = 91.0 (SE-CNN) AUC = 83.6 (SVM)
Le et al. [184]	2021	NSCLC-radiogenomics	LR / KNN / RF / XGBoost	*EGFR:* ACC = 77.8 *KRAS:* ACC = 83.3
Cheng et al. [187]	2021	Private (670 patients)	Pre-trained 3D DenseNet	*EGFR:* AUC = 76.0 ACC = 72.5 F-score = 71.3
Zhang et al. [186]	2021	Private (134 patients)	Logistic regression	*EGFR:* AUC = 78.0 *KRAS:* AUC = 81.0 *ERBB2:* AUC = 87.0 *TP53:* AUC = 84.0
Han et al. [185]	2021	Private (827 patients)	Logistic Regression	*EGFR:* AUC = 75.8 *ALK:* AUC = 73.9

ACC: Accuracy; AUC: area under the ROC curve; KNN: K-nearest neighbors; LR: logistic regression; MLP: multilayer perceptron; OR: odds ratio; RF: random forest; SCAV: selective class average voting; SE-CNN: squeezeand-excitation convolutional neural network; SVM: support vector machine; XGBoost: extreme gradient boosting.

**Table 7:** Overview of published studies regarding predictive models for gene mutation status based on nodule and extra nodule features (2017–2021).

Authors	Year	Dataset	Methods	Performance Results (%)
Gevaert et al. [71]	2017	Private (186 patients)	Decision Tree	*EGFR:* AUC = 89.0
Cao et al. [188]	2018	Private (156 patients)	Principal component analysis	*EGFR:* TPR = 72.3 TNR = 78.5
Rizzo et al. [189]	2019	Private (122 patients)	Univariate analysis	*EGFR:* AUC = 82.0 *KRAS:* AUC = 67.0
Pinheiro et al. [50]	2019	NSCLC-radiogenomics	Gradient tree boosting	*EGFR:* AUC = 74.6
Xiong et al. [190]	2019	Private (1010 patients)	ResNet 101	*EGFR:* AUC = 83.8
Silva et al. [191]	2021	LIDC-IDRI NSCLC-radiogenomics	Convolutional autoencoder	*EGFR:* AUC = 68.0
Morgado et al. [192]	2021	NSCLC-radiogenomics	LR, Elastic Net, Linear SVM, RBG SVM, RF, and XGBoost	*EGFR:* AUC = 73.7 (Linear SVM) AUC = 73.3 (Elastic Net) AUC = 72.5 (LR)

AUC: area under the ROC curve; LR: logistic regression; RF: random forest; SVM: support vector machine; TNR: true negative rate; TPR: true positive rate.

**Table 8:** Overview of published works regarding the prediction of PD-L1 expression status in lung cancer CT images (2017–2021).

Authors	Year	Dataset	Methods	Performance Results (%)
Toyokawa et al. [193]	2017	Private (394 patients)	Fisher’s exact test Univariate/multivariate LR (CT features)	PD-L1^+^ statistical association: (*p* < 0.01)—convergence, notching, spiculation, cavitation
Wu et al. [194]	2019	Private (350 patients)	Univariate/multivariate LR Fisher’s exact test Mann–Whitney *U* test	AUC = 78.3 TPR = 81.1 TNR = 64.1
Zhu et al. [195]	2020	Private (127 patients)	Univariate/multivariate LR 3D DenseNet	AUC = 78.0 ACC = 77.8 TPR = 77.8 TNR = 77.4
Jiang et al. [197]	2020	Private (399 patients)	Random forest Logistic regression	AUC = 97.0 (≥1%) AUC = 80.0 (≥50%)
Tian et al. [196]	2021	Private (939 patients)	Fully connected classifier	AUC = 76.0
Yang et al. [199]	2021	Private (200 patients)	Simple temporal attention (SimTA) module	AUC = 77.0 (SimTA_60_) AUC = 80.0 (SimTA_90_) AUC = 69.0 (RNN) AUC = 64.0 (Radiomics)
Jiang et al. [198]	2021	Private (125 patients)	Random forest Decision tree Logistic regression AdaBoost Support vector machine	(Internal validation) AUC = 96.0 TNR = 80.0 TPR = 98.5 (External validation) AUC = 85.0 TNR = 63.6 TPR = 91.3

ACC: accuracy; AUC: area under the ROC curve; LR: logistic regression; RNN: recurrent neural network; SimTAx: response prediction x days post immunotherapy; TNR: true negative rate; TPR: true positive rate.
